# A Comparative Study of Ferulic Acid on Different Monosaccharide-Mediated Protein Glycation and Oxidative Damage in Bovine Serum Albumin

**DOI:** 10.3390/molecules181113886

**Published:** 2013-11-11

**Authors:** Weerachat Sompong, Aramsri Meeprom, Henrique Cheng, Sirichai Adisakwattana

**Affiliations:** 1 Program in Clinical Biochemistry and Molecular Medicine, Department of Clinical Chemistry, Faculty of Allied Health Sciences, Chulalongkorn University, Bangkok 10330, Thailand; E-Mails: weerachat.tum@gmail.com (W.S.); meeprom.a@gmail.com (A.M.); 2 Department of Comparative Biomedical Sciences, School of Veterinary Medicine, Louisiana State University, Baton Rouge, LA 70803, USA; E-Mail: hcheng@vetmed.lsu.edu; 3 Research Group of Herbal Medicine for Prevention and Therapeutic of Metabolic Diseases, Chulalongkorn University, Bangkok 10330, Thailand; 4 The Medical Food Research and Development Center, Department of Nutrition and Dietetics, Faculty of Allied Health Sciences, Chulalongkorn University, Bangkok 10330, Thailand

**Keywords:** protein glycation, oxidative damage, ferulic acid, monosaccharides, advanced glycation end-products (AGEs), comparative study

## Abstract

Three dietary monosaccharides, (glucose, fructose, and ribose), have different rates of protein glycation that accelerates the production of advanced glycation end-products (AGEs). The present work was conducted to investigate the effect of ferulic acid (FA) on the three monosaccharide-mediated protein glycations and oxidation of BSA. Comparing the percentage reduction, FA (1–5 mM) reduced the level of fluorescence AGEs (F-AGEs) and N^ε^-(carboxymethyl) lysine (N^ε^-CML) in glucose-glycated BSA (F-AGEs = 12.61%–36.49%; N^ε^-CML = 33.61%–66.51%), fructose-glycated BSA (F-AGEs = 25.28%–56.42%; N^ε^-CML = 40.21%–62.91%), and ribose-glycated BSA (F-AGEs = 25.63%–51.18%; N^ε^-CML = 26.64%–64.08%). In addition, the percentages of FA reduction of fructosamine (Frc) and amyloid cross β-structure (Amy) were Frc = 20.45%–43.81%; Amy = 17.84%–34.54% in glucose-glycated BSA, Frc = 25.17%–36.92%; Amy = 27.25%–39.51% in fructose-glycated BSA, and Frc = 17.34%–29.71%; Amy = 8.26%–59.92% in ribose-glycated BSA. FA also induced a reduction in protein carbonyl content (PC) and loss of protein thiol groups (TO) in glucose-glycated BSA (PC = 37.78%–56.03%; TO = 6.75%–13.41%), fructose-glycated BSA (PC = 36.72%–52.74%; TO = 6.18%–20.08%), and ribose-glycated BSA (PC = 25.58%–33.46%; TO = 20.50%–39.07%). Interestingly, the decrease in fluorescence AGEs by FA correlated with the level of N^ε^-CML, fructosamine, amyloid cross β-structure, and protein carbonyl content. Therefore, FA could potentially be used to inhibit protein glycation and oxidative damage caused by monosaccharides, suggesting that it might prevent AGEs-mediated pathologies during diabetic complications.

## 1. Introduction

Protein glycation is a non-enzymatic reaction between the carbonyl groups of monosaccharides such as glucose, fructose, and ribose with the amino groups of proteins. The reaction initiates a complex cascade of repeated condensations, rearrangements, and oxidative modifications resulting in the reversible formation of structures called Schiff’s bases. This is followed by rearrangement to produce Amadori products like fructosamine [1,2]. Consequently, the Amadori products form cross-linked structures termed advanced glycation end-products (AGEs) [1,2,3]. AGEs can be classified into two major groups: fluorescent and crosslinking structures (pentosidine, crosslines, and imidazolones) and non-fluorescent and non-crosslinking structures (N^ε^-(carboxymethyl)lysine, N^ε^-CML) [4]. Accumulation of AGEs in body tissues plays a major role in the development of diabetic complications such as retinopathy, nephropathy, neuropathy, age-related diseases, atherosclerosis and Alzheimer’s disease [5,6,7,8]. Interestingly, a number of studies report that the rate of protein glycation depends upon the concentration and type of monosaccharide that enhances the generation of AGEs [9,10]. In this regard, these occurrences increase the risk for developing complications related to diabetes. Recently, considerable attention has been devoted to the search for phytochemical compounds that inhibit protein glycation. These compounds could aid in the prevention of complications during diabetes.

Ferulic acid (FA) possesses many pharmacological effects, especially anti-diabetic activity [11,12,13]. Furthermore, it inhibits AGE formation in physiological and food model systems [13,14,15]. However, the ability of FA to inhibit protein glycation induced by different monosaccharides has not been evaluated. In the present study, we investigated the inhibitory effect of FA on three different types of monosaccharide-induced protein glycation and oxidative damage using a bovine serum albumin (BSA) *in vitro* model. 

## 2. Results and Discussion

### 2.1. Effect of Ferulic Acid on Advanced Glycation End-Products (AGEs) and Amadori Products

**Figure 1:**
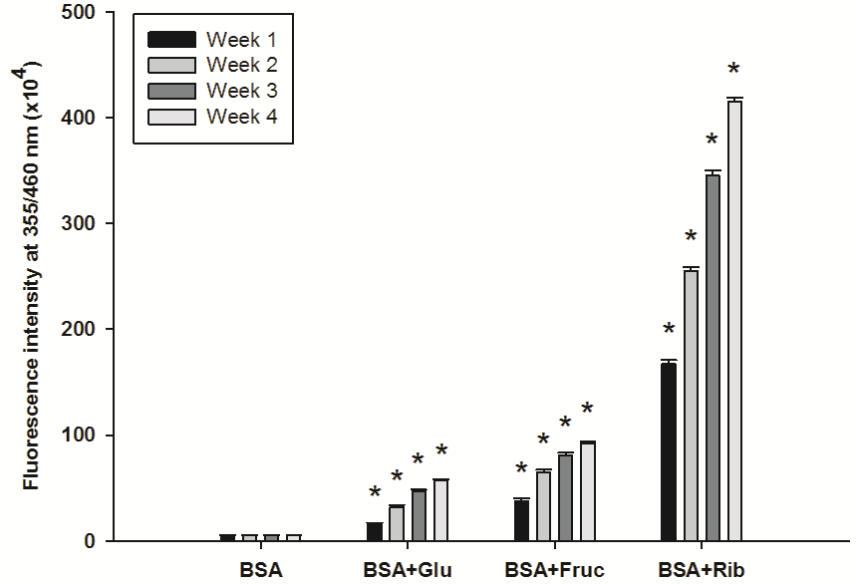
Time course of fluorescence intensity in BSA incubated with 500 mM glucose (Glu), 500 mM fructose (Fruc) or 100 mM ribose (Rib). The results are expressed as mean ± SEM (*n* = 3). * *p* < 0.05 when compared to BSA.

As shown in Figure 1, the fluorescence intensity of AGEs in BSA incubated with three types of monosaccharides during four weeks significantly increased throughout the incubation period. At week 4, the fluorescence intensity of glucose-, fructose-, and ribose-glycated BSA was 10.71-, 17.22-, and 77.23-fold higher than control non-glycated BSA. Protein glycation is a spontaneous reaction between monosaccharides and proteins that produces unstable Schiff bases that form Amadori products such as fructosamine, which is clinically used as an indicator for short-term control of blood sugars in diabetic patients [1,2]. This reaction generates irreversible heterogeneous products termed advanced glycation end products (AGEs) [2]. The rate of glycation is dependent on the concentration and type of monosaccharide [9,10]. All three monosaccharides exhibited protein cross-linking effects with different potency (glucose < fructose < ribose). Previous studies revealed faster accumulation of protein-bound fluorescence and oligomerization with fructose than glucose [16]. Moreover, the glycating ability of monosaccharides occur in the following increasing order: d-glucose < d-mannose < d-galactose < d-xylose < d-fructose < d-arabinose < d-ribose < 2-deoxy-D-ribose [17]. The highest glycating capability of d-ribose is explained by its planar structure causing the unstable aldofuranose ring to react with the amino groups [10]. 

**Figure 2:**
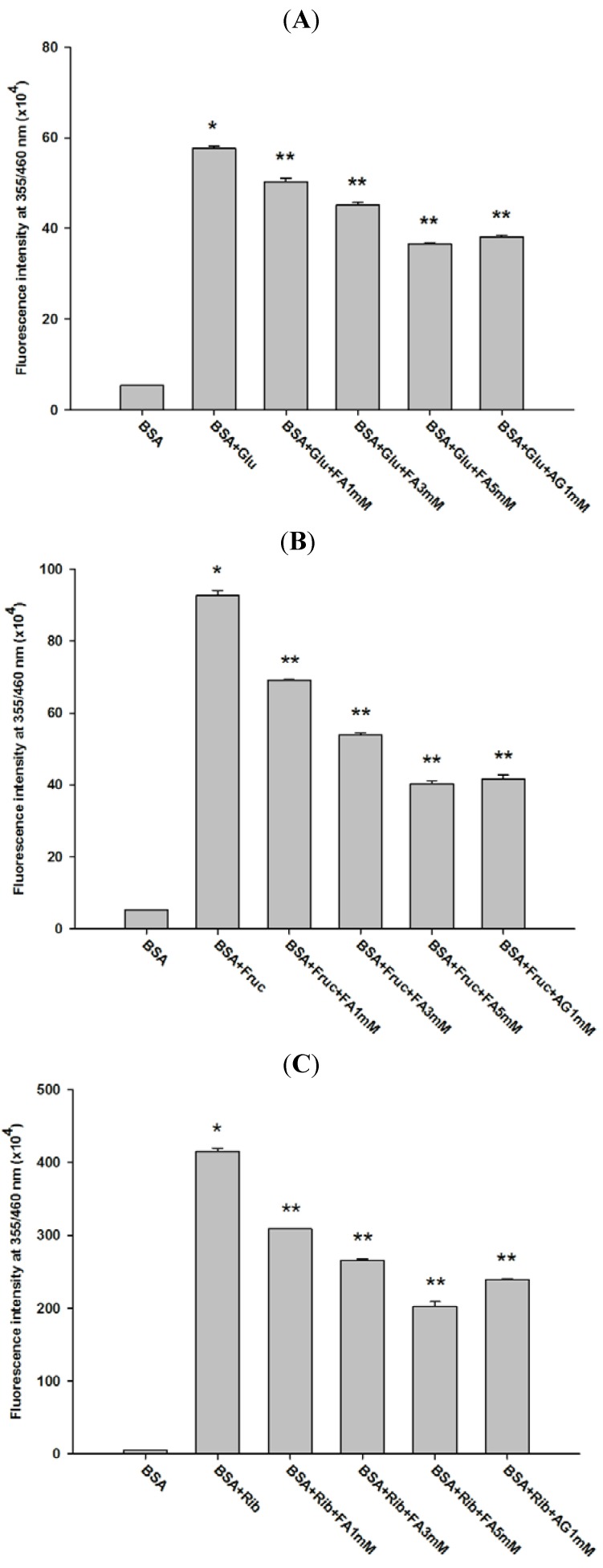
Effect of FA on the fluorescence intensity of BSA incubated with glucose (**A**), fructose (**B**), and ribose (**C**) at week 4. The results are expressed as mean ± SEM (*n* = 3). * *p* < 0.05 when compared to BSA, ** *p* < 0.05 when compared to BSA+glucose, BSA+fructose or BSA+ribose. AG was used as positive control.

Figure 2 shows the effect of FA on fluorescent AGE formation in glycated BSA after four weeks of incubation. The addition of FA (1, 3, and 5 mM) into the reaction mixtures significantly decreased the fluorescence intensity of glucose-glycated BSA (12.61%, 21.70%, and 36.49%), fructose-glycated BSA (25.28%, 41.79%, and 56.42%), and ribose-glycated BSA (25.63%, 35.91%, and 51.18%). Furthermore, AG (1 mM) significantly decreased the fluorescence intensity of glucose-glycated BSA (33.81%), fructose-glycated BSA (54.93%), and ribose-glycated BSA (42.31%). Comparing the percentages of reduction, FA inhibited more fructose-glycated BSA than glucose-glycated BSA. At the same concentration of 1 mM, the percentage inhibition of FA was less than AG in glucose-, fructose-, and ribose-glycated BSA. Silván *et al.* reported that FA markedly reduces the formation of N^ε^-CML and fluorescent AGEs during high fructose exposure [18]. Other studies revealed that lysine-524 is the major site of protein modification that leads to the loss of the corresponding unmodified tryptic peptide [19]. FA is also capable of binding to human serum albumin [20]. This binding causes structural changes and prevents BSA oxidation. Based on these findings, it can be hypothesized that FA binds to the same site of AGE formation in BSA that reduces glycation. Further studies are needed to test this hypothesis. 

Cinnamic acid and its derivatives are widely present in the diet, serving as the most abundant source of antiglycation agent [21,22,23]. In a previous report, cinnamic acid was the most effective inhibitor against fructose-mediated protein glycation [21]. The ability to inhibit protein glycation of cinnamic acid derivatives is associated with the presence of hydroxy and methoxy substituents in their structures. Caffeic acid, a naturally occurring cinnamic acid, inhibits the formation of AGEs induced by methylglyoxal [22]. Nevertheless, caffeic acid also exhibits the pro-oxidative activity which facilitates the progress of protein glycation, thereby promoting the production of AGEs [23]. We have raised the possibility of a protective effect of isoferulic acid, an isomer of ferulic acid, in glucose- and fructose-mediated protein glycation. The results show that isoferulic acid can exert antiglycation activity in a concentration-dependent manner [24].

**Figure 3:**
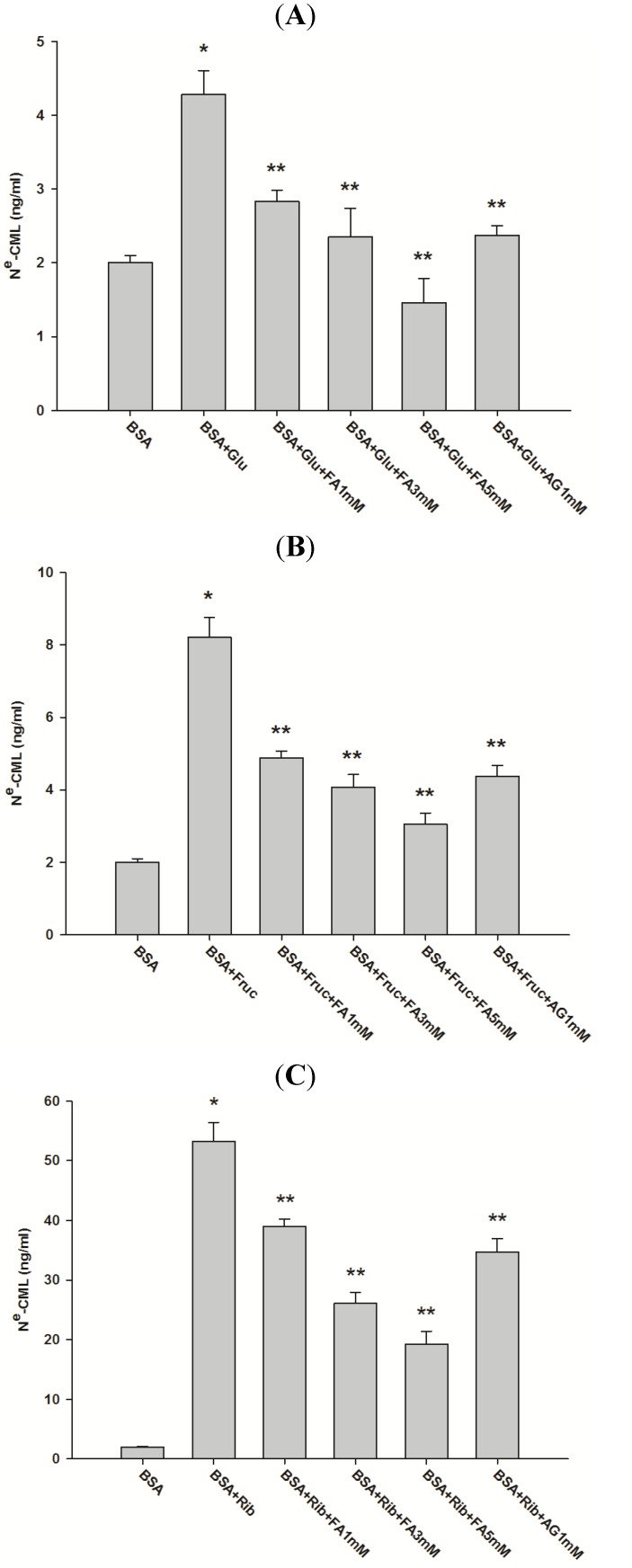
Effect of FA on non-fluorescence AGEs level (N^ε^-CML) in BSA incubated with glucose (**A**), fructose (**B**) and ribose (**C**) at week 4. The results are expressed as mean ± SEM (*n* = 3). * *p* < 0.05 when compared to BSA, ** *p* < 0.05 when compared to BSA+glucose, BSA+fructose or BSA+ribose. AG was used as positive control.

Figure 3 shows the effect of FA on N^ε^-CML level in glycated BSA after four weeks of incubation. The concentration of N^ε^-CML in glucose-, fructose-, and ribose-glycated BSA was 2.13-, 4.08- and 26.51-fold higher than non-glycated BSA. It was interesting to note that FA (1, 3, and 5 mM) significantly reduced the concentration of N^ε^-CML in glucose-glycated BSA (33.61%, 45.84%, and 66.51%), fructose-glycated BSA (40.21%, 50.39%, and 62.91%), and ribose-glycated BSA (26.64%, 51.05%, and 64.08%). These results suggest that FA can protect against advanced glycation end product formation. In addition, AG (1 mM) decreased the concentration of N^ε^-CML in glucose-, fructose-, and ribose-glycated BSA by 44.44%, 46.62%, and 34.83%, respectively. The results indicate that the percentage inhibition of FA (1 mM) was lower than the percentage inhibition of AG (1 mM) in glucose, fructose-, and ribose-glycated BSA.

**Figure 4:**
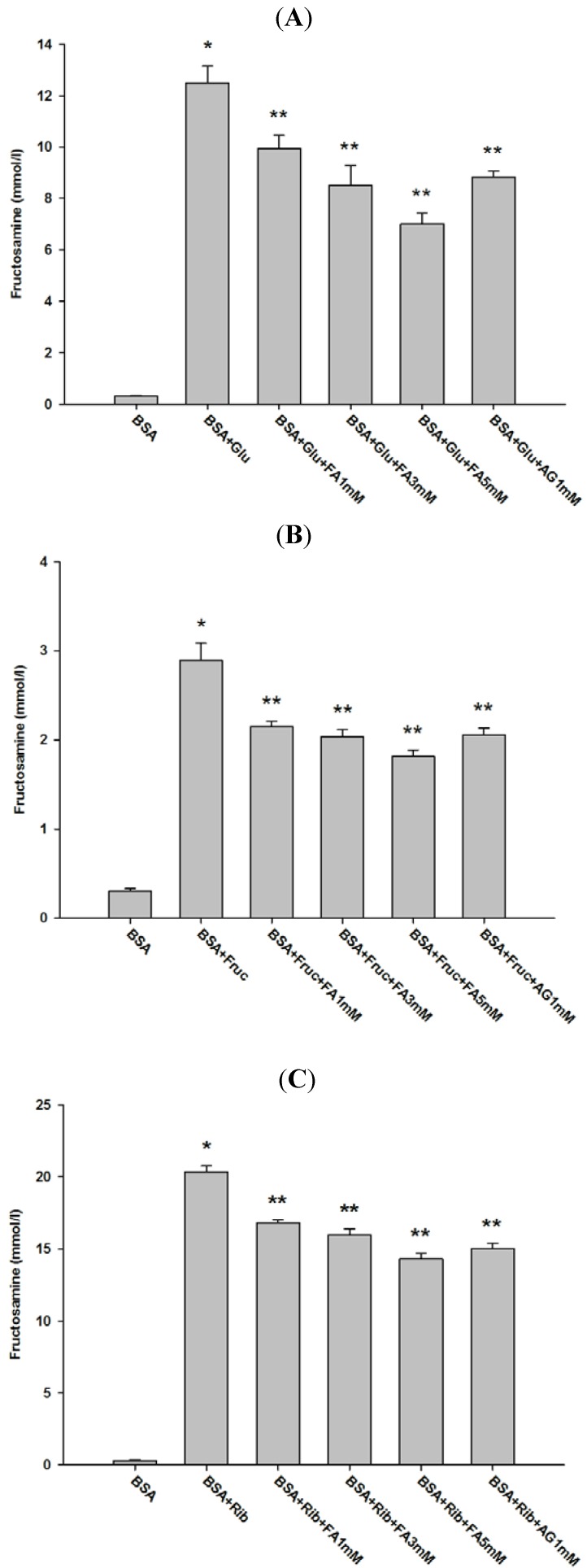
Effect of FA on fructosamine level in BSA incubated with glucose (**A**), fructose (**B**) and ribose (**C**) at week 4. The results are expressed as mean ± SEM (*n* = 3). * *p* < 0.05 when compared to BSA, ** *p* < 0.05 when compared to BSA+glucose, BSA+fructose or BSA+ribose. AG was used as positive control.

As shown in Figure 4, the level of fructosamine in BSA incubated with glucose, fructose, and ribose increased by 41.57-, 9.59-, and 68.12-fold respectively compared to non-glycated BSA. The results demonstrated that ribose exhibited the highest rate of fructosamine production among the three monosaccharides. When comparing the effect of fructose and glucose on protein glycation, many reports describe that fructose has greater capability to form fluorescent and non-fluorescent AGEs than glucose [24,25]. Consequently, this may suggest that fructose is much more prone to produce the Amadori product (fructosamine) than glucose. However, in our study, the level of fructosamine in fructose-glycated BSA was lower than in glucose-glycated BSA. 

This discrepancy can be explained by the limitations of the fructosamine assay. Non-enzymatic protein glycation contributes to the difference in structure of the aldehydic and ketonic Amadori products. The level of fructosamine is commonly determined through the reaction between glycation of amino groups in BSA and a choromogenic compound nitroblue tetrazolium (NBT) under alkaline conditions. The disadvantage of the NBT assay is that aldehydic Amadori products partially react with the NBT redox dyes [26]. This may be the reason for underestimating fructosamine levels in fructose-glycated BSA. 

Furthermore, FA (1–5 mM) showed a significant reduction in fructosamine levels in glucose-glycated BSA (20.45%–43.81%), fructose-glycated BSA (25.17%–36.92%), and ribose-glycated BSA (17.34%–29.71%). According to these results, it can be suggested that FA inhibits the formation of fluorescent and non-fluorescent AGEs associated with reduced fructosamine levels. In addition, AG (1 mM) demonstrated a significant reduction in fructosamine, since the percentages of fructosamine reduction of glucose-, fructose-, and ribose-glycated BSA were 29.09%, 28.56%, and 26.21%, respectively. Comparison of the percentage inhibition showed that FA (1 mM) was less effective than AG (1 mM) in the reduction of fructosamine in glucose-, fructose-, and ribose-glycated BSA. It is proposed that blockage of carbonyl or dicarbonyl groups in monosaccharides, Schiff’s bases or Amadori products can be used as a strategy to inhibit protein glycation [4]. AG acts as a carbonyl trapping agent by forming guanidine-dicarbonyl adducts, thereby reducing the number of free carbonyl groups in reducing sugars during the early stages of glycation [27]. 

### 2.2. Effect of Ferulic Acid on Protein Aggregation

**Figure 5:**
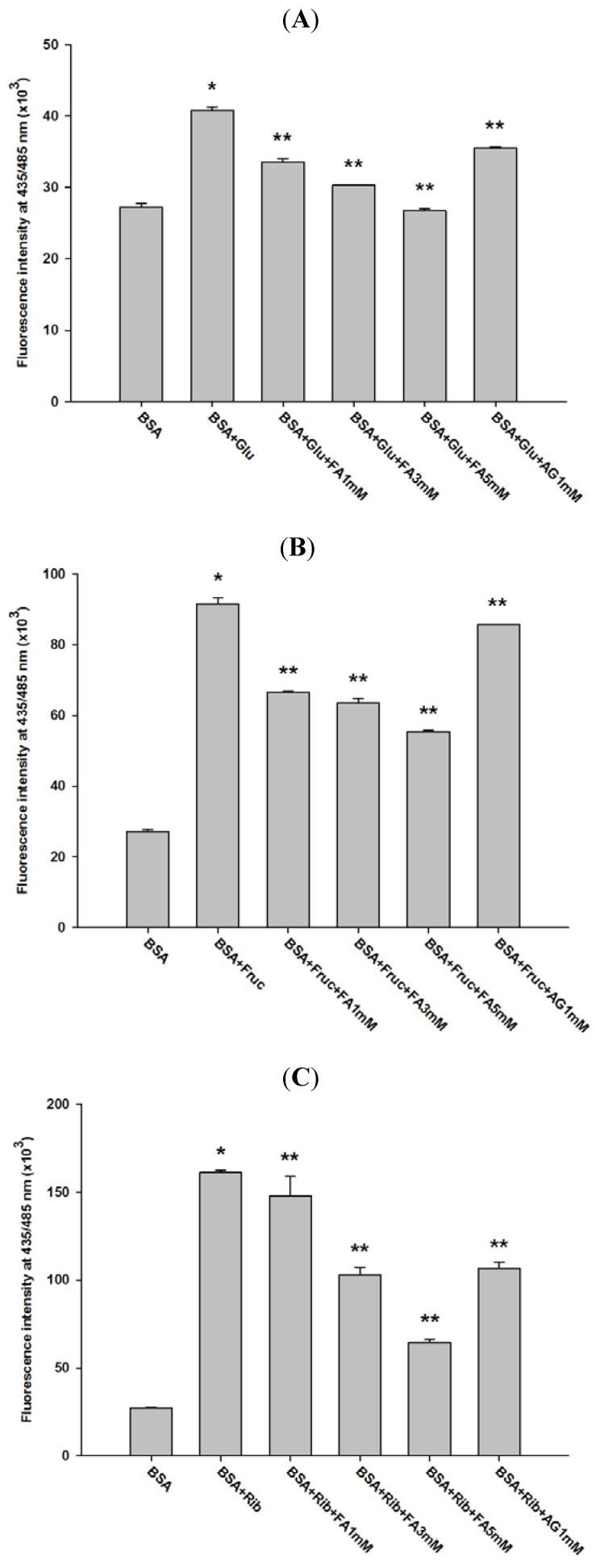
Effect of FA on amyloid cross β-structure levels in BSA incubated with glucose (**A**), fructose (**B**) and ribose (**C**) at week 4. The results are expressed as mean ± SEM (*n* = 3). * *p* < 0.05 when compared to BSA, ** *p* < 0.05 when compared to BSA+glucose, BSA+fructose or BSA+ribose. AG was used as positive control.

The levels of amyloid cross β-structures in BSA incubated with the three monosaccharides are presented in Figure 5. After four weeks of incubation, the fluorescence intensity of glucose-, fructose-, ribose-glycated BSA significantly increased to 1.50-, 3.37- and 5.92-fold over the fluorescence intensity of non-glycated BSA. The addition of FA into the reaction caused a significant decrease in amyloid cross β-structure levels in BSA, ranging from 17.84% to 34.54% (glucose), 27.25% to 39.51% (fructose), and 8.26% to 59.92% (ribose). A significant decrease in amyloid cross β-structure levels (12.87% in BSA+glucose, 6.39% in BSA+fructose, and 33.71% in BSA+ribose) was observed in the presence of AG (1 mM). Comparatively, the ability to reduce protein aggregation in glucose-glycated and fructose-glycated BSA by ferulic acid (1 mM) was higher than AG (1 mM). However, the percentage reduction of ribose-glycated BSA by FA was less than AG. 

Protein glycation is believed to be a key mechanism to accelerate the formation of protein aggregation and amyloid cross β-structures leading to altered protein structure and stability [28,29]. The long-term accumulation of amyloid cross β-structures in tissues and organs is linked to the progression of pancreatic islet amyloidosis which directly destroys β-cell and impairs insulin secretion [30,31]. The present findings demonstrate that FA reduces the formation of amyloid cross β-structures in BSA. This beneficial effect might help reduce the risk of developing diabetes complications.

### 2.3. Effect of Ferulic Acid on Protein Thiol Group and Protein Carbonyl Content

The effect of FA on protein thiol group content in BSA incubated with the three monosaccharides is shown in Table 1. The incubation of BSA with glucose, fructose, and ribose significantly reduced the level of protein thiol groups (TO) in BSA by 18.53%, 28.40%, and 45.09% compared to BSA alone. The results showed that FA (1–5 mM) inhibited TO loss in BSA+Glu (6.75%–13.41%), BSA+Fruc (6.18%–20.08%), and BSA+Rib (20.50%–39.07%). In addition, AG (1 mM) significantly inhibited TO loss in BSA by 17.50%, 29.91% and 64.95% in the presence of glucose, fructose, and ribose. These results suggest that FA is less effective in the prevention of TO loss compared to AG. The effect of FA on protein carbonyl content (PCO) in BSA incubated with three monosaccharides is shown in Table 1. These results demonstrated that PCO levels in glucose-, fructose-, and ribose-glycated BSA is approximately 8.14-, 11.95- and 28.10-fold greater than non-glycated BSA. Meanwhile, FA (1–5 mM) caused a significant reduction in the protein carbonyl content level in BSA+Glu (37.78%–56.03%), BSA+Fruc (36.72%–52.74%), and BSA+Rib (25.58%–33.46%). Similarly, a significant decrease in PCO level (42.75% in BSA+Glu, 40.57% in BSA+Fruc, and 31.31% in BSA+Rib) was observed in the presence of AG (1 mM). Comparing the percent reduction in PCO at concentration of 1 mM, FA was more effective than AG only against glucose-mediated protein glycation.

**Table 1:** Effect of FA on protein thiol group and protein carbonyl content in BSA incubated with glucose, fructose, and ribose at week 4. AG was used as positive control. The results are expressed as mean ± SEM (*n* = 3). * *p* < 0.05 when compared to BSA, ** *p* < 0.05 when compared to BSA+Glucose, BSA+Fructose or BSA+Ribose.

Experimental groups	Protein thiol group	Protein carbonyl content
	(nmol/mg protein)	(nmol/mg protein)
BSA	0.92 ± 0.03	0.26 ± 0.04
BSA+Glu	0.75 ± 0.02 *	2.03 ± 0.06 *
BSA+Glu+FA 1 mM	0.80 ± 0.02	1.26 ± 0.04 **
BSA+Glu+FA 3 mM	0.83 ± 0.02	1.07 ± 0.03 **
BSA+Glu+FA 5 mM	0.85 ± 0.03	0.89 ± 0.04 **
BSA+Glu+AG 1 mM	0.88 ± 0.02 **	1.16 ± 0.06 **
BSA+Fruc	0.66 ± 0.03 *	2.98 ± 0.08 *
BSA+Fruc+FA 1 mM	0.70 ± 0.03	1.88 ± 0.04 **
BSA+Fruc+FA 3 mM	0.74 ± 0.01	1.68 ± 0.06 **
BSA+Fruc+FA 5 mM	0.79 ± 0.02	1.41 ± 0.03 **
BSA+Fruc+AG 1 mM	0.85 ± 0.04 **	1.77 ± 0.06 **
BSA+Rib	0.50 ± 0.01 *	6.97 ± 0.09 *
BSA+Rib+FA 1 mM	0.61 ± 0.02	5.19 ± 0.09 **
BSA+Rib+FA 3 mM	0.66 ± 0.05 **	4.93 ± 0.07 **
BSA+Rib+FA 5 mM	0.70 ± 0.02 **	4.64 ± 0.06 **
BSA+Rib+AG 1 mM	0.83 ± 0.02 **	4.79 ± 0.06 **

Thiol groups of proteins are a general target to determine the protein oxidation during glycation [32,33]. Cysteine and methionine are particularly prone to oxidative attack by free radical species such as superoxide and hydroxyl radicals. The direct oxidation of amino acid (Lys, Arg, Thr) or secondary reaction of amino acid residues (Cys and His) with reactive carbonyl compounds can produce the formation of PCO derivatives [34]. Hence, the protein carbonyl content is commonly used as a marker for protein oxidative damage [35]. The formation of AGEs is a multifactorial process. One of the mechanisms of AGEs formation and protein damage is through the production of reactive oxygen species (ROS). It is well established that protein glycation continually generates superoxide anions from early glycation products that include 1,2- and 2,3-enolization of the Schiff's base and oxidation of the enolate anion [36,37]. In addition, the Amadori product or Schiff's base undergoes fragmentation through reactive oxygen species-mediated reactions to generate short-chain carbohydrate intermediates, which alter lysine and arginine residues to produce AGEs. According to the formation of non-fluorescent and non-crosslinking structures so far, hydroxyl radicals generated by Fenton reactions between Fe^2+^ and Amadori product-derived endogenous H_2_O_2_ causes oxidative cleavage of Amadori compounds into N^ε^-CML [38]. This mechanism is involved in the defense against free radical attack on proteins [4]. Like many polyphenols, FA exhibits antioxidant activity by scavenging superoxide and hydroxyl radicals, suggesting that inhibition of protein glycation and AGE formation may be related to its antioxidant activity [39,40]. The results showed that FA can be as effective as AG for suppression of protein glycation. Unfortunately, AG was discontinued in Phase III clinical trials possibly due to its toxicity [41]. FA present in fruits and vegetables is considered to be a non-toxic compound that can be absorbed and metabolized in the body. Data on the bioavailability of FA ranges from 0.4% to 98% [42]. Based on the current knowledge, FA could be a promising inhibitor of AGE formation. 

### 2.4. Correlation Coefficients between Glycation and Protein Oxidation

Table 2 shows the Pearson’s coefficients (*r*) with the correlation between protein glycation and protein oxidation of BSA incubated with the three monosaccharides and FA. 

**Table 2:** Pearson’s correlation coefficients of fluorescence AGEs (F-AGEs), N^ε^-CML, fructosamine (Frc), amyloid cross β-structure (Amy), protein carbonyl content (PCO), and protein thiol group (TO) level in BSA incubated with the three types of monosaccharides and FA.

	F-AGEs	N^ε^-CML	Frc	Amy	PCO	TO
F-AGEs	-	0.987 *	0.826 *	0.904 *	0.978 *	−0.826 *
N^ε^-CML	-	-	0.806 *	0.919 *	0.956 *	−0.806 *
Frc	-	-	-	0.572 *	0.787 *	−0.512 *
Amy	-	-	-	-	0.896 *	−0.876 *
PCO	-	-	-	-	-	−0.845 *
TO	-	-	-	-	-	-

* Significant at *p* < 0.001.

**Figure 6:**
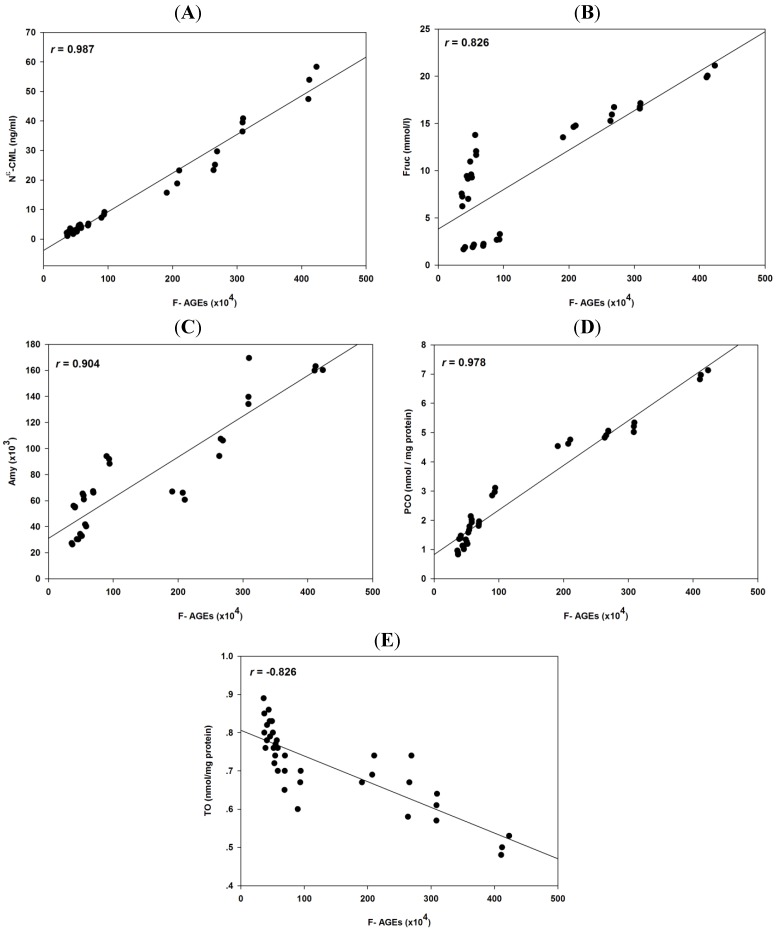
Correlations between fluorescent AGEs (F-AGEs), N^ε^-CML, fructosamine (Fruc), amyloid cross β-structure (Amy), protein carbonyl content (PCO), and thiol group (TO) in the BSA containing glucose, fructose, and ribose and various concentrations of FA (1–5 mM).

The level of non-fluorescent AGEs (N^ε^-CML), fructosamine, amyloid cross β-structure, and protein carbonyl content exhibited a positive correlation with the level of fluorescent AGEs (Figure 6). This suggests that the reduction in fluorescent AGEs in glucose-, fructose-, and ribose-glycated BSA by FA was directly proportional to the level of non-fluorescent AGEs (N^ε^-CML), fructosamine, amyloid cross β-structure, and protein carbonyl content. Conversely, there was a negative correlation between the level of fluorescent AGEs and protein thiol group, suggesting that FA caused a decrease in the levels of fluorescent AGEs in glucose-, fructose-, and ribose-glycated BSA that was inversely proportional to the level of protein thiol group in glycated BSA. A positive correlation between the level of non-fluorescent AGEs (N^ε^-CML), fructosamine, amyloid cross β-structure, and protein carbonyl content and a negative correlation between the level of non-fluorescent AGEs (N^ε^-CML) and protein thiol group were observed with all three monossaccharides. Interestingly, the level of fructosamine exhibited a positive and negative correlation with the levels of amyloid cross β-structure and protein thiol groups, respectively.

## 3. Experimental

### 3.1. Chemicals

Bovine serum albumin (BSA, fraction V), ferulic acid (FA, 4-hydroxy-3-methoxycinnamic acid), aminoguanidine hydrochloride (AG), d-ribose, nitroblue tetrazolium (NBT), 1-deoxy-1-morpholino-d-fructose (1-DMF), guanidine hydrochloride, thioflavin T, 5,5'-dithiobis(2-nitrobenzoic acid) (DTNB) and l-cysteine were purchased from Sigma-Aldrich Co. (St. Louis, MO, USA). d-Glucose, d-fructose and 2,4-dinitrophenyl hydrazine (DNPH) were purchased from Ajax Finechem (Taren Point, Australia). Trichloroacetic acid (TCA) was purchased from Merck (Darmstadt, Germany). OxiSelect™ N^ε^-(carboxymethyl) lysine (N^ε^-CML) ELISA kit was obtained from Cell Biolabs (San Diego, CA, USA). All other chemicals and solvents used in this study were analytical grade.

### 3.2. *In Vitro* Glycation of Bovine Serum Albumin (BSA) Induced by Monosaccharides

Glycation of BSA was done according to a previous method with minor modifications [43]. Briefly, 10 mg/mL BSA was incubated with 500 mM glucose, 500 mM fructose and 100 mM ribose in 100 mM phosphate buffered-saline (pH 7.4) containing 0.02% sodium azide at 37 °C for four weeks in the absence or presence of FA and AG (a positive control). Before incubation, FA (final concentration: 1, 3, and 5 mM) and AG (final concentration: 1 mM) were added into the reaction mixtures. A final concentration of 4% dimethylsulfoxide (DMSO) was used as solvent for the study. Samples were stored at −20 °C before analysis.

### 3.3. Determination of Advanced Glycation End-Products (AGEs)

The formation of fluorescent AGEs was measured using a spectrofluorometer (Wallac 1420 Victor^3^ V, PerkinElmer, Santa Clara, CA, USA). The fluorescence intensity was measured at an excitation wavelength of 355 nm and emission wavelength of 460 nm. The concentration of non-fluorescent AGEs (N^ε^-(carboxymethyl) lysine, N^ε^-CML), a major non-fluorescent AGE structure, was measured by using an enzyme linked immunosorbant assay (ELISA) kit according to the manufacturer’s protocol. The concentration of N^ε^-CML was calculated by using a N^ε^-CML-BSA standard curve.

### 3.4. Determination of Fructosamine (Amadori Products)

The level of Amadori products was measured as fructosamine by using nitroblue-tetrazolium (NBT) dye according to a previous method with minor modifications [44]. Glycated BSA was incubated with 0.5 mM NBT in 100 mM carbonate buffer (pH 10.4) at 37 °C. The absorbance was measured at 530 nm. The level of fructosamine was calculated using the different absorption at the time point of 10 and 15 min, and compared to 1-deoxy-1-morpholino-d-fructose (1-DMF) as the standard. 

### 3.5. Determination of Protein Aggregation

Amyloid cross β-structure, a common marker for protein aggregation was measured using the thioflavin T assay according to a previous method with minor modifications [45]. Glycated BSA was incubated with 32 µM thioflavin T in 100 mM phosphate buffered-saline (pH 7.4) at room temperature for 60 min. The fluorescence intensity was measured at an excitation wavelength of 435 nm and emission wavelength of 485 nm.

### 3.6. Determination of Protein Thiol Group

Protein thiol group was measured according to Ellman’s assay with minor modifications [46]. Glycated BSA was incubated with 5 mM 5,5'-dithiobis(2-nitrobenzoic acid) (DTNB) in 100 mM phosphate buffered-saline (pH 7.4) at room temperature for 15 min and the absorbance measured at 412 nm. The level of protein thiol group was calculated from a standard curve prepared using l-cysteine. The results were expressed as nmol l-cysteine/mg protein. 

### 3.7. Determination of Protein Carbonyl Content

Protein carbonyl content, a common marker for protein oxidative damage, was measured according to a previous method with minor modifications [47]. Glycated BSA was incubated with 10 mM 2,4-dinitrophenylhydrazine (DNPH) in 2.5 M HCl at room temperature for 60 min. Afterwards, it was precipitated by 20% (w/v) trichloroacetic acid (TCA), left on ice for 5 min, and centrifuged at 10,000 *g* at 4 °C for 10 min. The pellet was washed three times using 1:1 (v/v) ethanol-ethyl acetate mixture. The final pellet was dissolved in 6 M guanidine hydrochloride. The absorbance was recorded at 370 nm. The level of protein carbonyl content was calculated by using an absorption coefficient of 22,000 M^−1^cm^−1^. The results were expressed as nmol carbonyls/mg protein. 

### 3.8. Statistical Analysis

Data were expressed as mean ± standard error of mean (SEM). The statistical significance of the results was evaluated using one-way ANOVA. Tukey’s HSD test was used to analyze the differences between the means. *p* < 0.05 was considered to be statistically significant.

## 4. Conclusions

For the first time, FA was evaluated for its inhibitory effects against three monosaccharide-mediated protein glycation reactions and oxidative damage of BSA. Using *in vitro* models, FA markedly inhibited protein glycation and oxidative damage in BSA induced by glucose, fructose, and ribose. Consequently, FA decreased the formation of fluorescent AGEs, non-fluorescent AGEs and fructosamine associated with the reduction of protein aggregation and protein carbonyl content. It also prevented the loss of protein thiol groups. Given the ability of FA to inhibit monosaccharide-mediated protein glycation and oxidation, our findings could be potentially useful in the prevention of AGE-mediated pathologies during diabetic complications.
